# A modified sequence capture approach allowing standard and methylation analyses of the same enriched genomic DNA sample

**DOI:** 10.1186/s12864-018-4640-y

**Published:** 2018-04-13

**Authors:** Lisa Olohan, Laura-Jayne Gardiner, Anita Lucaci, Burkhard Steuernagel, Brande Wulff, John Kenny, Neil Hall, Anthony Hall

**Affiliations:** 1 Institute of Integrative Biology, University of Liverpool, Crown Street, Liverpool, UK; 2 Earlham Institute, Norwich research Park, Norwich, UK; 3 John Innes Centre, Norwich Research Park, Norwich, UK; 4 University of East Anglia, Norwich, UK

**Keywords:** Sequence capture, Methodology, Combining genotype and epi-type analysis

## Abstract

**Background:**

Bread wheat has a large complex genome that makes whole genome resequencing costly. Therefore, genome complexity reduction techniques such as sequence capture make re-sequencing cost effective. With a high-quality draft wheat genome now available it is possible to design capture probe sets and to use them to accurately genotype and anchor SNPs to the genome. Furthermore, in addition to genetic variation, epigenetic variation provides a source of natural variation contributing to changes in gene expression and phenotype that can be profiled at the base pair level using sequence capture coupled with bisulphite treatment. Here, we present a new 12 Mbp wheat capture probe set, that allows both the profiling of genotype and methylation from the same DNA sample. Furthermore, we present a method, based on Agilent SureSelect Methyl-Seq, that will use a single capture assay as a starting point to allow both DNA sequencing and methyl-seq.

**Results:**

Our method uses a single capture assay that is sequentially split and used for both DNA sequencing and methyl-seq. The resultant genotype and epi-type data is highly comparable in terms of coverage and SNP/methylation site identification to that generated from separate captures for DNA sequencing and methyl-seq. Furthermore, by defining SNP frequencies in a diverse landrace from the Watkins collection we highlight the importance of having genotype data to prevent false positive methylation calls. Finally, we present the design of a new 12 Mbp wheat capture and demonstrate its successful application to re-sequence wheat.

**Conclusions:**

We present a cost-effective method for performing both DNA sequencing and methyl-seq from a single capture reaction thus reducing reagent costs, sample preparation time and DNA requirements for these complementary analyses.

**Electronic supplementary material:**

The online version of this article (10.1186/s12864-018-4640-y) contains supplementary material, which is available to authorized users.

## Background

Bread wheat has a large complex allohexaploid genome that is 17GB in size and made up from three progenitor genomes (AABBDD). This size makes whole genome resequencing costly [1]. Therefore, a number of reduced representation sequencing approaches exist that make re-sequencing cost effective. These include approaches such as: Restriction site Associated DNA sequencing or RAD-seq [2], involving digesting DNA with restriction enzymes and sequencing a tag for each resulting fragment; transcriptome sequencing, where we sequence cDNA generated from mRNA [3]; sequence capture, the capture and sequencing of DNA fragments by the hybridization of genomic DNA with synthesized probes. With a high-quality draft wheat genome now available, it is possible to design capture probe sets for tiling evenly across the genome and to use them to accurately genotype and anchor SNPs and CNVs to the genome [4].

In addition to genetic variation, epigenetic variation also provides a source of natural variability contributing to changes in gene expression and phenotype. The most common form of DNA methylation is 5-methylcytosine, an epigenetic mark found throughout the genome of most eukaryotic organisms. Cytosine methylation has been implicated with orchestrating the structure and function of the genome, regulating chromatin and gene expression and it is found in plants in the context of CG, CHG and CHH [5, 6]. It is thought that cytosine methylation may be important for plants, providing a mechanism for rapidly adapting to environmental change.

Bisulphite treatment deaminates unmethylated cytosines resulting in conversion from a cytosine to a uracil residue. Therefore, bisulphite treatment in combination with sequencing can identify methylated cytosine residues, an approach termed methyl-seq [7]. Previously, we used methyl-seq in combination with sequence capture to survey the epigenome in hexaploid bread wheat [8]. An important question now is to understand how methylation varies across a globally diverse collection of wheat germplasm adapted to specific local agricultural niches. However, to apply methyl-seq to this kind of dataset you ideally require both DNA sequence data and bisulphite treated sequence data for each wheat accession otherwise C-T SNPS will be incorrectly classified as unmethylated cytosine sites.

Here, we describe a new wheat capture probe set that is tiled across the hexaploid bread wheat genome. We present a method, based on Agilent SureSelect Methyl-Seq, that will use a single capture assay as a starting point that is sequentially split and used for both DNA sequencing and methyl-seq. We validate the approach by comparing it to standard SureSelect and Methyl-seq sequencing datasets. We benchmark the approach with the reference accession Chinese Spring and demonstrate its utility with an accession from the Watkins collection.

## Results

### Our methodology has no detrimental effect on capture efficiency

Using our custom probe set, we initially follow a SureSelect Methyl-Seq library preparation and hybridisation protocol, however we divide the sample immediately after capture and, using two parallel custom protocols, we can take one aliquot through Illumina paired-end sequencing and the second aliquot through bisulphite conversion and Illumina paired-end sequencing (see Methods). In order to assess the quality of the sequencing data generated we also performed standard non-divided SureSelect and SureSelect Methyl-Seq enrichments followed by sequencing and compared the output. This splitting after capture “dual-purpose” methodology allows us to directly compare the genotype and epi-type of the same DNA sample.

**Fig. 1:**
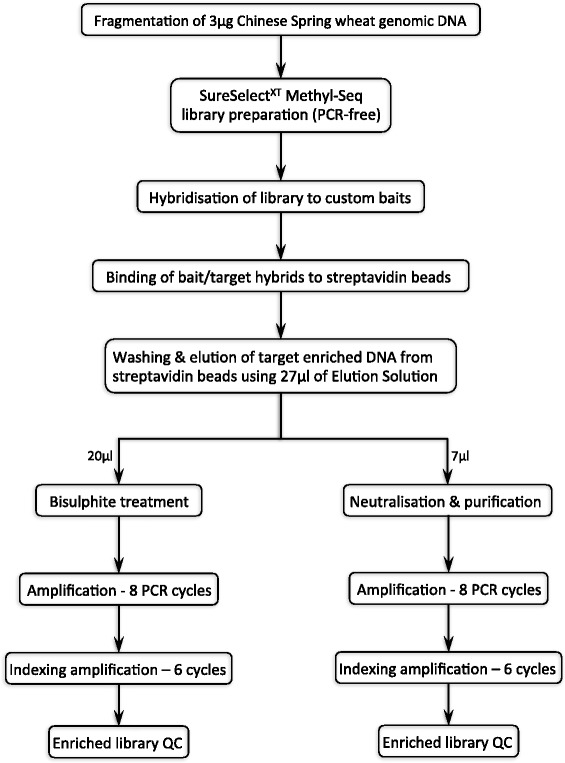
Workflow of the modified sequence capture method. Following fragmentation of the genomic DNA, a SureSelect Methyl-Seq library was constructed and hybridised to custom baits. Bait/target hybrids were bound to streptavidin beads, which were then washed to remove non- specifically bound DNA fragments. Target enriched DNA was eluted from the streptavidin beads and the eluate divided; ~ 3/4 of the eluate was bisulphite converted and then amplified, ~ 1/4 was neutralised, purified and then amplified. The quality of the purified libraries was assessed prior to sequencing

For the first enrichment, (i) a standard SureSelect library was prepared; this is referred to as non-bisulphite treated full (NBTF). For the second enrichment, (ii) a SureSelect Methyl-Seq library was prepared; this is referred to as bisulphite treated full (BTF). For the third enrichment (Fig. 1), (iii) a Sureselect Methyl-Seq library was again prepared and hybridised as usual but the enriched DNA was eluted, divided and bisulphite converted according to our modified dual-purpose methodology; this is subsequently referred to as bisulphite treated split (BTS). The remaining eluted DNA was neutralized, amplified and sequenced according to our parallel modified protocol; this is subsequently referred to as non-bisulphite treated split (NBTS).

**Fig. 2:**
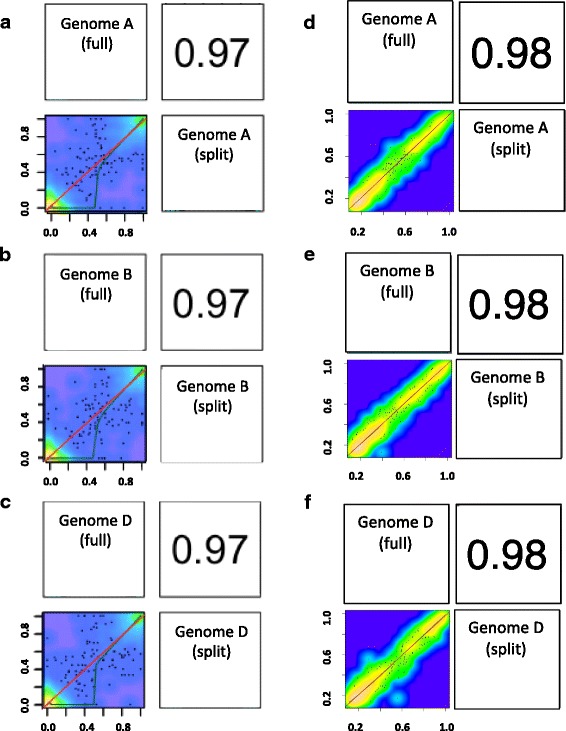
Methylkit Pearson correlation coefficient computations to compare methylation and SNPs between split and non-split samples. Figures demonstrate comparisons of methylation levels across the bisulphite and non-bisulphite treated samples at positions that are associated with **a** sub-genome A **b** sub-genome B and **c** sub-genome D. Comparisons of SNPs using allele frequencies were also computed for the same comparisons and are shown for SNPs in **d** sub-genome A **e** sub-genome B and **f** sub-genome D. Individual samples are labeled diagonally with an axis through the middle of the plot that acts as a mirror image division; comparative correlation plots lie to the left of the axis at the intersection between the two samples, with the corresponding correlation co-efficient for the plot to the left of the axis at the intersection between the two samples

Using paired-end sequencing reads to extend into the regions surrounding the capture probes, the mapped space exceeds the capture probe set design of 12 Mb by more than 4× and 3× in the data from the non-bisulphite treated and bisulphite-treated samples, respectively. Looking at the non-bisulphite treated datasets, full and split enrichments were equivalent with neither more than 1.2% from their average sequencing depth of 35.7×, across 51.7 Mb of the extended reference bait sequence (Table 1). The depth of coverage across the probe set was summarised for the non-bisulphite treated samples across pseudo chromosomal molecules that were generated using POPseq data [9] and coverage was relatively consistent with most falling into the range 5-70× (Additional file 1: Figure S1). On average, only 6% of baits exceeded 2× the average depth of coverage i.e. excessively high depth and ~ 4% showed a low average coverage less than 5×. The vast majority (97.4%) of SNPs were conserved between the full and split samples at positions that were mapped to a minimum depth of 10× per sample (948,282). Furthermore, Pearson correlation plots demonstrate high SNP comparability between samples with correlation coefficients consistently at 0.98 when sub-genomes are compared between the full and split datasets (Fig. 2). For the non-bisulphite treated datasets there were 49.4 million sequencing reads in the non-split sample and after mapping and duplicate removal 32% of reads were aligned. Similarly, in the split sample, there were 44.8 million sequencing reads, of which, an equivalent 36.6% of reads were aligned after duplicate removal (Table 1).

**Table 1:** Mapping statistics for the reference sequence

Sample	% of reads aligned pre-filtering	Average % coverage per ref. contig	Average depth of coverage per ref. contig	Number of ref. contigs mapped	% of ref. contigs mapped	Base-space mapped (bp)
BTF	24.2	59.6	30.7	82,873	99.6	39,868,184
BTS	24.2	59.3	30.5	82,862	99.6	39,602,695
NBTF	73.3	70.7	36.9	83,107	99.9	48,808,952
NBTS	72.8	77.9	34.5	82,999	99.7	54,641,687

Detailing the mapping output statistics for the two enriched wheat DNA samples, NBTF and BTF (non-bisulphite treated and bisulphite treated) that were taken through separate capture reactions and the two samples that were split and one bisulphite treated while the other was non-bisulphite treated after a single capture (NBTS and BTS). Mapping statistics are in relation to the 82.5 Mb mapping reference

The full and split bisulphite treated samples had an average depth of coverage of 30.6×, with neither more than 0.1% from this average, across 39.7 Mb of the extended reference bait sequence. For the bisulphite treated datasets there were 49.9 million sequencing reads in the non-split sample and after mapping and duplicate removal 21.8% of reads were aligned and available for analysis. In the split sample, there were 50 million sequencing reads, of which, a highly comparable 21.5% of reads remained for analysis after mapping and duplicate removal (Table 1).

For the bisulphite treated full and split samples, differential methylation between the A, B and D sub-genomes was recorded using the tool methylKit to identify a minimum difference of 25% and *p* < 0.01 (see Methods). 239,100 residues were available for comparison between the two samples i.e. residues with a depth of 5× or more per sub-genome in both samples. Of these 239,100 residues, only 0.006% showed methylation differences. This methylation between the samples is more similar than that seen between biological replicate samples in our previous studies (< 0.09% difference observed [8]) and highlights our maintained capability to confidently define methylation patterns even after sample splitting. Furthermore, Pearson correlation plots demonstrate high comparability between samples with correlation coefficients consistently at 0.97 when sub-genomes are compared between the full and split datasets (Fig. 2).

Mapping the bisulphite treated sequencing reads to the non-methylated chloroplast genome was used to assess bisulphite conversion efficiency i.e. the percentage of cytosine bases that were successfully bisulphite converted [10, 11]. While we did not enrich for chloroplast DNA specifically, a small proportion of our reads are carryover DNA equivalent to low coverage shotgun sequencing of total wheat DNA, therefore a subset of these off-target sequences map to the wheat chloroplast genome. Mapping statistics are shown in Table 2 where a consistently high level of coverage was gained (> 350×) and highly comparable conversion efficiencies of 98.73% for the full sample and 98.82% for the split sample were observed.

**Table 2:** Mapping statistics for the chloroplast genome

Sample	% cytosine bases successfully converted	Average depth of coverage	% of chloroplast genome mapped	Base-space mapped (bp)
BTF	98.73	391.5	99.73	114,672
BTS	98.82	386.6	99.75	114,691

Detailing the mapping output statistics for the bisulphite treated enriched wheat DNA sample, BTF, that was taken through an individual capture reaction and the sample that was split after capture and bisulphite treated (BTS). Mapping statistics are in relation to the chloroplast genome mapping reference

### Demonstrating the utility of this method and capture probe set using a diverse wheat landrace

Our split after capture protocol was followed exactly as for Chinese Spring, however this time using a random line from the Watkins bread wheat diversity collection (accession 1190103). This resulted in the generation of a bisulphite treated split (BTS) and non-bisulphite treated split (NBTS) library for the Watkins accession. Enriched libraries were sequenced on an Illumina HiSeq 4000 generating 2 × 125 bp paired-end reads and sequencing reads were aligned to the mapping reference as per the methodology for Chinese Spring.

The split bisulphite treated sample had an average depth of coverage of 42.4× across 42.3 Mb of the extended reference bait sequence while the split non-bisulphite treated sample had an average depth of coverage of 66.7× across 51.1 Mb (Table 3). This is highly comparable to the mapping coverage generated by Chinese Spring (39–54 Mbp mapped), therefore the capture probe set can successfully enrich diverse wheat landraces that are thought to show a high SNP density compared to the reference accession Chinese Spring that is the basis of the capture design.

**Table 3:** Mapping statistics for the reference sequence (Watkins line 1190103)

Sample	Average % coverage per ref. contig	Average depth of coverage per ref. contig	Number of ref. contigs mapped	% of ref. contigs mapped	Base-space mapped (bp)
BTS	64.8	42.4	81,634	98.1	42,266,334
NBTS	76.3	66.7	82,970	99.7	51,111,629

Detailing the mapping output statistics for the two Watkins wheat 1190103 samples that were split and one bisulphite treated while the other was non-bisulphite treated after a single capture (NBTS and BTS). Mapping statistics are in relation to the 82.5 Mb mapping reference

SNPs were defined for the non-bisulphite treated dataset yielding 2,022,551 SNPs at a minimum of 10×. Of these SNPs, 672,949 were C➔T or G➔A SNPs that could be incorrectly classified as unmethylated cytosine sites if they were unidentified. Furthermore, 779,185 SNPs resulted in a C/G residue in the Watkins line where there was an A/T previously and these represent key missed opportunities where accession specific methylation, from accession specific cytosine residues that deviate from the reference sequence, may not have been previously analysed and therefore identified. The bisulphite treated sequencing data enables the analysis of 5,962,239 cytosines that show sequencing coverage at a minimum of 10× that is sufficient for accurate methylation calls; correction of the reference sequence for this Watkins line using the 672,949 C/G➔T/A SNPs has the potential to eliminate up to 11.3% of these calls that were likely to be inaccurate and correction of the reference sequence using the 779,185 A/T➔C/G SNPs would increase the cytosine set for analysis by approximately 1/5th.

This analysis demonstrates the utility of the capture probe set to enrich a diverse wheat accession that is likely to show a high SNP density compared to Chinese Spring while also quantifying the extent of the problems that we may encounter by not genotyping while we epi-type i.e. define how many residues could be given false positive methylation calls due to SNPs.

**Fig. 3:**
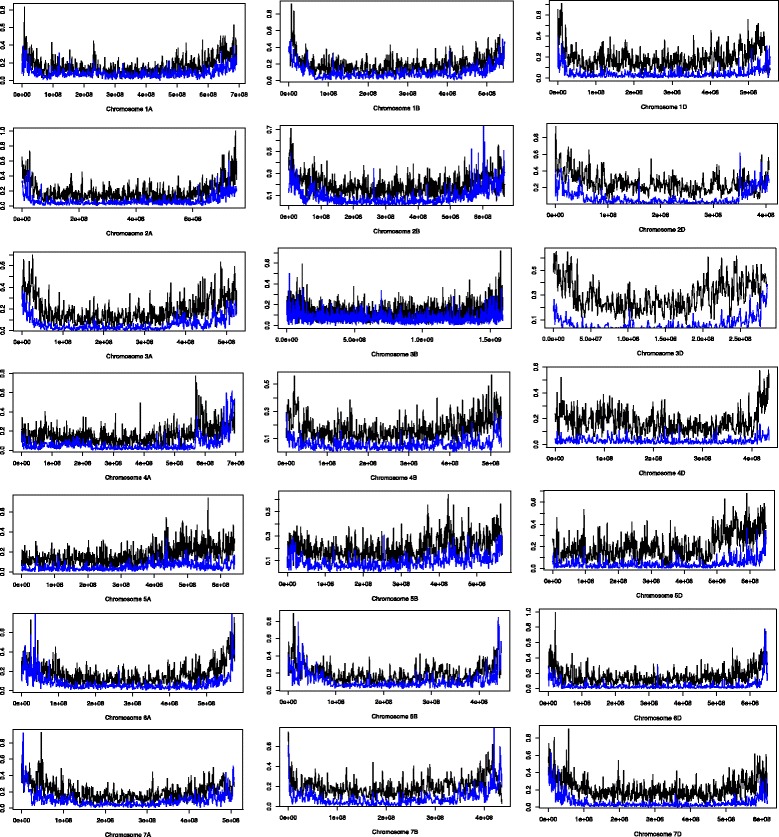
Representation of capture sequence data for Watkins accession 1190103 across the wheat chromosomes. Normalized frequency plots of SNP (blue) and cytosine positions for which DNA methylation information is available (black) per 1Mbp window across each wheat chromosomal pseudomolecule. Normalization of frequencies is to a scale of 0–1. Pre-normalization SNP maximum frequency was 1759 and minimum frequency was 0, cytosine maximum was 9214 and minimum was 0

The distribution of SNPs and DNA methylation information for the Watkins accession was assessed across the chromosomal pseudomolecules [9] (Fig. 3). It is clear that the capture probe set generates informative sequence data that is distributed across the genome with a bias towards genic regions that are more common towards chromosome ends. 58.3% of SNP/DNA methylation information is found at genes with 7.3% in promoters. Furthermore, 97.4% of the 2,022,551 SNP sites and 99.8% of the 5,962,239 cytosines with DNA methylation information in Watkins accession 1190103 show sufficient coverage (minimum 10X) also in Chinese Spring. This gives the potential for large scale in-depth comparative analyses between enriched accessions.

## Discussion

Here, we describe a new wheat capture probe set that is tiled across the hexaploid bread wheat genome. This capture probe set is evenly tiled across the genome and enriches typically over 4× the probe design space. It can be effectively utilised to survey and observe genome wide trends in wheat from a genotypic or epigenetic perspective. Furthermore, it can successfully enrich DNA from a diverse wheat accession from the Watkins landrace diversity collection despite it being designed based on the reference variety Chinese Spring.

Using this probe set, we present a method, based on Agilent SureSelect Methyl-Seq, that will use a single capture assay as a starting point that is sequentially split and used for both DNA sequencing and methyl-seq. This method is applicable to any organism of interest and therefore has a much wider usage potential than the use on wheat that we demonstrate here as an example. Understanding variation across populations is a common scientific question and we want to understand how methylation changes across a globally diverse collection of wheat germplasm using methyl-seq therefore we will require both DNA sequence data and bisulphite treated sequence data for each wheat accession otherwise CG-TA SNPS will be incorrectly classified as unmethylated cytosine sites. Furthermore, looking at a diverse landrace from the Watkins collection, this problem had the potential to affect 11.3% of sites, therefore this issue is of high priority to address. Moreover, correction of the reference sequence using A/T➔C/G SNPs could increase the cytosine set for analysis by 1/5th yielding further benefit to analyses.

With a single Agilent SureSelect capture reaction costing in excess of £500 (probe set plus capture reagents based on purchasing a set of 16), by utilising a single capture for both genotype and epigenetic analysis, we can cut these considerable costs. These savings are in addition to the reduction in staff labour costs associated with performing a lower number of capture reactions. Furthermore, there is an additional benefit to performing only one capture reaction to generate genotype and epigenetic information if the DNA quantity that is available for an individual sample is restricted.

Methyl-seq protocols from Agilent’s companies such as NimbleGen use an approach where bisulphite conversion is carried out pre-capture. Since bisulphite conversion severely diminishes DNA concentration, this allows users to maximize input into expensive capture reactions by treating as much DNA as possible. However, this necessitates the development of a fully converted and fully non-converted probe for each region of DNA that is more amenable to mammalian systems where methylation has largely been previously profiled and only symmetrical CpG methylation is present typically in so-called highly methylated islands. In many accessions of wheat, and in other plants, the methylation profile is mainly unknown, methylation can exist in both symmetrical and non-symmetrical forms (CpG, CHG and CHH), partial methylation in a region is common and methylation is not always in islands. This makes the design of probes for treatment-pre-capture difficult and incorrectly designed probes could introduce unwanted bias into captures. Here we treat post-capture, this allows the same probes to be used for genotyping and methyl-seq analysis. Our split after capture protocol has been carefully developed to deal with the low DNA concentrations associated with bisulfite treatment post-capture.

When using approaches where bisulphite conversion is carried out pre-capture, software has been developed to allow sample genotyping directly from bisulphite treated sequencing data and although this would reduce costs, removing the need for both DNA sequence and bisulphite treated sequence data, this method depends heavily on having sequence information for both DNA strands in the effort to discriminate C-T SNPs and in plants, has all the previously described problems with conversion-pre-capture. Here, we profile methylation in only one strand of DNA, and as such this requirement for both strands would double the probe capture space and increase sequencing and probe costs. Furthermore, due to the high complexity of genotyping directly from the bisulphite treated sequencing data, such methodologies are highly error prone with reported false positive/false negative SNP calling rates ranging from 15% to upwards of 50% [12]. We, therefore present a gold standard methodology to ensure highly accurate SNP calls and following on from this high-quality methylation calls.

## Conclusions

If we wish to accurately profile DNA methylation in diverse wheat lines, then it is advantageous to also generate genotype information. Here, we describe a new capture probe set that is tiled across the hexaploid bread wheat genome and can be effectively utilised to survey genome wide trends in wheat from a genotypic or epigenetic perspective. Furthermore, we present a cost-effective method for performing both DNA sequencing and methyl-seq from a single capture reaction thus significantly reducing reagent costs and DNA requirements.

## Methods

### Design of the wheat capture probe set

Probes were designed to capture a subset of wheat genes totaling 36 Mb; 12 Mb from each of the three sub-genomes of wheat (Additional file 2: Figure S2). The design space was a subset of the 110 Mb of assembled wheat genic sequence previously used for a NimbleGen (Roche) exome capture probe set. The 110 Mb was derived from the gene-rich regions of hexaploid bread wheat that had been processed to remove repetitive sequence, remove chloroplast and mitochondrial sequence, collapse redundant sequence and collapse homoeologous genes into one representative sequence [4]. Initially, 120 bp sequences were tiled across the 110 Mb of genic sequence at 40 bp intervals resulting in 2.3 million potential probe sequences. These probes were then annotated with the following information: (i) % alignment to the International Wheat Genome Sequencing Consortium (IWGSC) reference sequence (positional information and gene annotations were also recorded); (ii) number of homoeologous and varietal SNPs (utilising IWGSC, CerealsDB and the wheat ancestral genomes), to allow discrimination between the wheat sub-genomes and to capture diversity, respectively; and (iii) average depth of coverage of the region obtained in previous sequence capture experiments using NimbleGen probes [4, 13, 14]. These annotations were used to rank the probes and the ‘best’ 100,000 were selected for the capture probe set. In addition to the genome wide tiling, for genes identified as associated with drought tolerance (Additional file 3: Table S1) [15–18] and the NB-ARC conserved domains of nucleotide-binding site leucine-rich repeat (NBS-LRR) disease resistance genes [19], 120-mer probes were tiled end-to-end across these key sequences to ensure that they were enriched effectively. Finally, a bias for even tiling of probes across the chromosomes was implemented, i.e. where possible there was one of the 100,000 probes per assembled contig that was thought to represent a gene with additional bias for available surrounding sequence to facilitate effective mapping.

The 120 bp RNA capture probe or ‘bait’ sequences were uploaded to Agilent eArray (online custom microarray design tool) to allow submission for manufacture. Bait ‘boosting’ was selected to permit excess unused design space (less than 1 Mb in this case) to be filled with repeat sequences of baits predicted to perform less efficiently i.e. those with an above average GC content are ‘boosted’ to ultimately gain even depth of sequence coverage across the target region.

### Genomic DNA extraction and QC

Genomic DNA was extracted from the areal tissue of 10-day old Chinese Spring wheat seedlings grown at 22 °C using a DNeasy Plant Mini kit (Qiagen) according to the manufacturer’s instructions. The DNA was quantified using a Qubit double-stranded DNA high sensitivity assay kit and Qubit fluorometer (Invitrogen). 100 ng of DNA was analysed electrophoretically on a 1% agarose gel alongside HyperLadder 1 kb (Bioline) to determine DNA integrity. This indicated that the extracted DNA was high molecular weight, with minimal degradation and no evidence of RNA contamination. DNA purity was assessed by obtaining the 260/280 and 260/230 absorbance ratios on a NanoDrop ND-1000 spectrophotometer.

### Genomic DNA fragmentation

Three 3 μg aliquots of the same genomic DNA were each made up to a total volume of 130μl with 10 mM Tris-HCl, pH 8.0. After mixing, each was transferred to a separate Covaris AFA microTUBE with pre-split snap-cap (Product number 520045) and sheared to an average size of approximately 200 bp using a Covaris S2 focused-ultrasonicator (duty cycle 10%, intensity 5, 200 cycles per burst for 6 × 60s using frequency sweeping). The size distribution of the fragmented DNA was assessed with an Agilent 2100 Bioanalyser using a high sensitivity DNA chip. Each DNA aliquot was then purified using 1.4 × AxyPrep Mag PCR Clean-Up beads (Axygen) with two 70% ethanol washes (400 μl) and elution in 50 μl of nuclease-free water (Ambion). Each aliquot of purified fragmented DNA was used as input material for standard SureSelect library preparation or SureSelect Methyl-Seq library preparation as described below.

### Standard SureSelect target enrichment

A standard SureSelect library was constructed and hybridised essentially as described by the manufacturer in the SureSelect^XT^ Target Enrichment System for Illumina Multiplexed Sequencing Protocol; Version B.1, December 2014 (Agilent Manual Part Number G7530–90000), except all purification steps were carried out using AxyPrep Mag PCR Clean-Up beads instead of AMPure XP beads, since the former were more economical. Briefly, following end-repair, 3′-adenylation and paired-end adapter-ligation, 15 μl (approximately half) of the adapter-ligated DNA was used as template in the pre-capture PCR with 5 cycles of amplification. The purified pre-capture library was quantified by Qubit double-stranded DNA high sensitivity assay and the quality assessed on a Bioanalyser high sensitivity DNA chip. The DNA fragment size peak was 245 bp and the average fragment size was approximately 300 bp.

Based upon the concentration obtained by Qubit quantification, 750 ng of the pre-capture library was dehydrated until just dry by centrifugation under vacuum at 30°C in an Eppendorf Concentrator 5301. The DNA was then re-dissolved in 3.4 μl of nuclease-free water and hybridised for approximately 20 h at 65 °C to 5 μl of biotinylated custom SureSelect cRNA baits targeting the desired 12 Mb of wheat sequence. The hybridisation was set up according to the Agilent protocol using 2 μl of 25% RNase Block since the target was > 3.0 Mb. At the end of the hybridisation, bait/target hybrids were bound to 50 μl of Dynabeads MyOne Streptavidin T1 magnetic beads (Invitrogen). Following post-capture washing, the target-enriched library was resuspended in 30 μl of nuclease-free water and stored at − 20 °C for ~ 84 h.

Approximately half (14 μl) of the bead-bound library was subsequently amplified with a primer containing an 8 bp index using 10 PCR cycles. The purified captured library was quantified by Qubit double-stranded DNA high sensitivity assay and the size distribution ascertained by analysis on a Bioanalyser high sensitivity DNA chip. The library peaked at 287 bp with an average fragment size of approximately 330 bp.

### SureSelect methyl-Seq target enrichment

A SureSelect Methyl-Seq library was prepared and hybridised by following the manufacturer’s instructions in the SureSelect^XT^ Methyl-Seq Target Enrichment System for Illumina Multiplexed Sequencing Protocol; Version C.0, January 2015 (Agilent Manual Part Number G7530–90002). The guide was followed from end-repair onwards, and again AMPure XP beads were replaced by AxyPrep Mag PCR Clean-Up beads. Quality assessment after end-repair was omitted since the DNA had already been analysed immediately after fragmentation.

Following methylated adapter ligation, the DNA was purified with elution in 25 μl of nuclease-free water. The DNA size distribution was assessed on a Bioanalyser high sensitivity DNA chip and the library found to have a peak size of approximately 250 bp and an average fragment size of 300 bp. DNA concentration was determined by Qubit double-stranded DNA high sensitivity assay. The total yield of methylated adapter-ligated DNA was approximately 1.3 μg and all of this was concentrated as previously described, reconstituted in 3.4 μl of nuclease-free water and then used in the hybridisation step. The latter was conducted as described in the Agilent Manual but the Human methyl-seq capture library baits were substituted by 5 μl of our 12 Mb wheat-specific SureSelect baits. After approximately 20 h at 65 °C, bait/target hybrids were bound to streptavidin beads. Following post-capture washing, the bead-bound captured DNA was eluted with 20 μl of SureSelect Elution Solution and bisulphite-treated using an EZ DNA Methylation-Gold Kit (Zymo Research) according to the instructions in the Agilent protocol. At this point, the bisulphite-converted and desulphonated library was stored at − 20 °C for ~ 84 h. The library was then amplified using 8 cycles for the first PCR and 6 cycles for the indexing PCR with an indexing prime containing an 8 bp index. The final library was quantified by Qubit double-stranded DNA high sensitivity assay and analysed electrophoretically on a Bioanalyser high sensitivity DNA chip. The library fragments had an average size of 360 bp, with a peak at approximately 300 bp.

### Modified SureSelect protocol

A SureSelect Methyl-Seq library was constructed and hybridised exactly as described in the previous section. Based on quantification obtained by Qubit double-stranded DNA high sensitivity assay, 1.2 μg of methylated adapter-ligated DNA was obtained at the end of pre-capture library preparation. As previously, the DNA fragments peaked around 250 bp and the average fragment size was 300 bp when examined on a Bioanalyser high sensitivity DNA chip. The hybridisation was set up as outlined above using all of the pre-capture library as input. After the ~ 20 h 65 °C hybridisation, bait/target hybrids were bound to streptavidin beads and standard post-capture washing was carried out. This time, 27 μl of SureSelect Elution Solution was used to elute the target enriched DNA from the streptavidin beads. The beads were mixed with the Elution Solution and incubated at room temperature for 20 min, as instructed in the Agilent protocol. After this time, the beads and Elution Solution were separated using a DynaMag-2 magnet (ThermoFisher Scientific) and the supernatant divided into a 20 μl aliquot and a 7 μl aliquot – each being transferred to a separate 1.5 ml Eppendorf tube. 7 μl of SureSelect Neutralisation Solution was added to the 7 μl aliquot of eluted DNA; after mixing by brief vortexing, the DNA was placed on ice. The 20 μl aliquot of eluted DNA underwent bisulphite conversion and desulphonation according to the instructions in the Agilent protocol. During the bisulphite treatment (2.5 h at 64 °C, followed by 4 °C hold), the other, neutralised aliquot was purified using 1.8 × AxyPrep Mag PCR Clean-Up beads. For this, 16 μl of nuclease-free water was added to the 14 μl mixture, bringing the volume up to 30 μl. 54 μl of AxyPrep Mag PCR Clean-Up beads were then added and a standard clean-up was carried out with two 70% ethanol washes (350 μl) and elution with 19 μl of nuclease-free water. At this point, the target-enriched, purified DNA (~ 19 μl), and the enriched, bisulphite converted and desulphonated DNA (~ 20 μl) were both frozen at − 20 °C for ~ 84 h. The samples were then amplified in parallel according to the Agilent protocol. Although the two samples had been treated differently, the same amplification reagents and PCR cycling conditions were used. So, for the first PCR, each reaction contained 30 μl of nuclease-free water, 50 μl of SureSelect Methyl-Seq PCR Master Mix, 1 μl of Methyl-Seq PCR1 Primer F, 1 μl of Methyl-Seq PCR1 Primer R and 18 μl of enriched DNA (bisulphite-treated or non-treated). The following cycling conditions were used: 95 °C for 2 min, followed by 8 cycles of 95 °C for 30 s, 60 °C for 30 s and 72 °C for 30 s. A final extension of 72 °C for 7 min was used followed by a hold at 4 °C until further processing. The reactions were purified using 180 μl of AxyPrep Mag PCR Clean-Up beads with two 70% ethanol washes (450 μl) and elution with 21 μl of nuclease-free water. Each eluate was then used as template in the final indexing amplification where each reaction contained 25 μl of SureSelect Methyl-Seq PCR Master Mix, 0.5 μl of SureSelect Methyl-Seq Indexing Primer Common, 5 μl of Indexing Primer (containing an 8 bp index) and 19.5 μl of enriched amplified library (bisulphite-treated or non-treated). The cycling conditions were: 95 °C for 2 min, followed by 6 cycles of 95 °C for 30 s, 60 °C for 30 s and 72 °C for 30 s. A final extension of 72 °C for 7 min was used followed by a hold at 4 °C. The reactions were purified using 90 μl of AxyPrep Mag PCR Clean-Up beads with two 70% ethanol washes (450 μl) and elution with 24 μl of nuclease-free water. The final libraries were quantified by Qubit double-stranded DNA high sensitivity assay and analysed electrophoretically on a Bioanalyser high sensitivity DNA chip. The bisulphite-treated library peaked around 300 bp with an average fragment size of 347 bp. The non-treated library peaked at 390 bp with an average size of 421 bp.

### Illumina sequencing

All four of the libraries (two bisulphite-treated and two non-treated) were sequenced together with four other libraries of the same type. So, the eight libraries were pooled in equimolar amounts based on the Qubit and Bioanalyser data. The pool was further purified using 1.8 × AxyPrep Mag PCR Clean-Up beads. The size of the final pool was assessed on a Bioanalyser high sensitivity DNA chip and the DNA concentration was determined initially by Qubit double-stranded DNA high sensitivity assay, and then by qPCR, using an Illumina library quantification kit (KAPA) on a Roche LightCycler 480 II system. Sequencing was carried out on an Illumina HiSeq 2500, using version 4 chemistry, generating 2 × 125 bp paired-end reads.

### Mapping reference sequence

The 12 Mb of probe sequences align uniquely to 52,143 of the IWGSC reference gene contigs yielding partial representation of each. Utilising paired-end sequencing reads it is possible to extend the 120 bp sequence that is captured by each probe to include surrounding regions. In previous studies this resulted in up to a 4× extension of coverage from the initial capture probe set. As such, in this case we anticipated capturing up to 48 Mb per wheat sub-genome i.e. 144 Mb overall. This necessitated a reference sequence that was constructed using the probes plus surrounding contiguous DNA sequence. These extended reference contigs ranged from 360 bp–13,168 bp with a median length of 783 bp. Therefore, in this study the total size of the mapping reference was ~ 82.5 Mb per sub-genome.

### Standard mapping pipeline

All mapping analyses of non-bisulphite treated samples were carried out using BWAmem (version 0.7.10). Paired-end reads were mapped as fragment reads due to short reference contigs and only unique best mapping hits were taken forward [20]. Mapping results were processed using SAMtools; any non-uniquely mapping reads, unmapped reads, poor quality reads (< 10) and duplicate reads were removed [21]. SNP calling in diploid datasets was carried out using the GATK Unified genotyper (after Indel realignment), which was used with a minimum quality of 50 and filtered using standard GATK recommended parameters, a minimum coverage of 5 and homozygous SNPs only were selected [22]. For polyploid datasets SAMtools mpileup was implemented with the SNP caller VarScan, to identify positions containing an alternate allele, with a minimum coverage of 5, an average mapping quality above 15 and a MAF of greater than 0.1 [23].

### Mapping of bisulphite treated DNA samples

The sequencing datasets for the samples were mapped to the extended probe sequence using Bismark, an aligner and methylation caller designed specifically for bisulphite treated sequence data. Sequencing reads were mapped as fragment reads rather than paired-end; a mismatch number of 3 was used and the non-directional nature of the library was specified [24]. The Bismark methylation extractor tool was then used to identify all cytosine residues within the mapping and categorize the reads mapping to them as un-methylated or methylated at that position while also detailing which type of potential methylation site was present (CHH, CHG or CpG). The mapping results were also processed for SNP calling using the standard polyploid pipeline described above.

### Determining a reference homoeologous SNP list

A reference homoeologous SNP list was determined across the 82.5 Mb mapping reference using the same methods detailed by Gardiner et al. [8]. Firstly, the wheat ancestral genomes were aligned to the 82.5 Mb reference to identify homoeologous SNPs directly. Secondly, non-bisulphite treated Chinese Spring sequencing reads were aligned to the IWGSC reference sequence to determine a genome of origin (only perfect and unique hits to one or two genomes were used). These genome assigned reads were then aligned to our single 82.5 Mb reference sequence, which is representative of the 3 sub-genomes, allowing the discrimination of homoeologous SNP positions. SNP calling for polyploid datasets was carried out as previously described. Using genome assigned reads allowed us to match up the alleles at SNP locations with the contributing wheat sub-genome to define an additional homoeologous SNP list.

### Association of cytosine residues with the reference homoeologous SNP list

SNP positions were identified in the enriched hexaploid wheat bisulphite treated sequencing dataset using the standard polyploid pipeline. Reads mapping to these SNP positions therefore have sufficient depth and average mapping quality overall and one or more alternate allele present. Those positions that could also be found in the homoeologous SNP list were selected for further analysis i.e. homoeologous SNPs within the treated data. Any sequencing read with a mapping quality over 20, containing a cytosine residue methylation status calculated by Bismark, plus a homoeologous SNP allele, can be identified. Its SNP allele can be matched to a sub-genome therefore associating methylation status of that cytosine residue with a wheat sub-genome. For each cytosine position a summary of the number of reads hitting it for each sub-genome and whether or not these reads are methylated can be produced.

### Implementation of methylkit

The software methylKit [25] was used to identify regions of differential methylation. Our summary of each cytosine position plus the number of reads hitting it for each sub-genome and whether or not these reads are methylated can be formatted and used directly as input for such analysis. Variation or differential methylation was recorded between the split and non-split bisulphite treated samples per sub-genome of wheat i.e. pairwise comparisons were between sub-genome A-A, B-B and D-D. Due to the use of pairwise comparisons the Fisher’s exact test was used to discriminate statistically significant differences (*p* < 0.01 and methylation difference of ≥50%).

### Construction of pseudo chromosomes from capture design contigs

We made use of 21 wheat chromosomal pseudomolecules that were created by organising and concatenating the IWGSC CSS assemblies using POPSEQ data [9]. BLASTN was used to place the extended probe sequences onto these chromosomal pseudomolecules (E-value cutoff 1e-5, minimum sequence identity 90 and minimum length of 100 bp) [26]. Relative positions for the capture design contigs along the chromosomal pseudomolecules could then be used to order them into our POPSEQ based pseudo-chromosomes. We desired 7 POPSEQ based pseudo-chromosomes, as per our capture probe set, that were representative of the 21 wheat chromosomes. Therefore the order of the capture design contigs along genome B’s chromosomal pseudomolecules 1–7 was preferentially utilised since the greatest number of contigs could be aligned to these sequences and therefore included (83%).

## Additional files

Additional file 1: **Figure S1.** Depth of coverage summarised for the non-bisulphite treated samples per extended bait sequence reference contig. Reference extended bait sequence contigs here are organized using POPseq chromosomal pseudomolecules. a) Displays data for the NBTS sample and b) displays data for the NBTF sample. (PDF 1425 kb)

Additional file 2: **Figure S2.** Design of the 12 Mbp wheat gene capture array. The 110 Mbp design target sequence for the capture probe set is as described by Gardiner et al. (Gardiner et al.*,* 2015). The RNA baits for this SureSelect Methyl-Seq Target Enrichment system are all 120 bp in length, unique, non-repetitive and are evenly placed across the available wheat genic target sequence according to the design illustrated. (PDF 187 kb)

Additional file 3: **Table S1.** Drought tolerance associated genes. 120-mer probes were tiled end-to-end across these genes of particular interest [15–18]. (PDF 81 kb)

## Abbreviations

Bp: Base pair
Mbp: Million base pairs
SNP: Single nucleotide polymorphism

## References

[1] Clavijo BJ (2017). An improved assembly and annotation of the allohexaploid wheat genome identifies complete families of agronomic genes and provides genomic evidence for chromosomal translocations. Genome Res.

[2] Baird NA (2008). Rapid SNP discovery and genetic mapping using sequenced RAD markers. PLoS One.

[3] De Wit P (2015). SNP genotyping and population genomics from expressed sequences – current advances and future possibilities. Mol Ecol.

[4] Gardiner LJ (2014). Using genic sequence capture in combination with a syntenic pseudo genome to map a deletion mutant in a wheat species. Plant J.

[5] Zhang X (2006). Genome-wide high-resolution mapping and functional analysis of DNA methylation in Arabidopsis. Cell.

[6] Cokus SJ (2008). Shotgun bisulphite sequencing of the Arabidopsis genome reveals DNA methylation patterning. Nature.

[7] Darst RP, Ausubel FM (2010). Bisulfite sequencing of DNA. Current protocols in molecular biology.

[8] Gardiner L-J (2015). A genome-wide survey of DNA methylation in hexaploid wheat. Genome Biol.

[9] Chapman JA (2015). A whole-genome shotgun approach for assembling and anchoring the hexaploid bread wheat genome. Genome Biol.

[10] Fojtová M (2001). Cytosine methylation of plastid genome in higher plants. Fact or artefact?. Plant Sci.

[11] Lister R (2008). Highly integrated single-base resolution maps of the epigenome in Arabidopsis. Cell.

[12] Gao S (2015). BS-SNPer: SNP calling in bisulfite-seq data. Bioinformatics.

[13] The International Wheat Genome Sequencing Consortium (IWGSC) (2014). A chromosome-based draft sequence of the hexaploid bread wheat (Triticum aestivum) genome. Science.

[14] Wilkinson PA (2009). CerealsDB 2.0: an integrated resource for plant breeders and scientists. BMC Bioinformatics.

[15] Liang Y (2011). Prediction of drought-resistant genes in Arabidopsis thaliana using SVM-RFE. PLoS One.

[16] Uga Y (2013). Control of root system architecture by DEEPER ROOTING 1 increases rice yield under drought conditions. Nat Genet.

[17] Huang D (2008). The relationship of drought-related gene expression in Arabidopsis thaliana to hormonal and environmental factors. J Exp Biol.

[18] Hu H, Xiong L (2014). Genetic engineering and breeding of drought-resistant crops. Annu Rev Plant Biol.

[19] Steuernagel B (2016). Rapid cloning of disease-resistance genes in plants using mutagenesis and sequence capture. Nat Biotechnol.

[20] Li H, Durbin R (2009). Fast and accurate short read alignment with burrows-wheeler transform. Bioinformatics.

[21] Li H (2009). The sequence alignment/map format and SAMtools. Bioinformatics.

[22] McKenna A (2010). The genome analysis toolkit: a MapReduce framework for analyzing next-generation DNA sequencing data. Genome Res.

[23] Koboldt DC (2012). VarScan 2: somatic mutation and copy number alteration discovery in cancer by exome sequencing. Genome Res.

[24] Krueger F, Andrews SR (2011). Bismark: a flexible aligner and methylation caller for bisulfite-Seq applications. Bioinformatics.

[25] Akalin A (2012). methylKit: a comprehensive R package for the analysis of genome-wide DNA methylation profiles. Genome Biol.

[26] Altschul SF, Gish W, Miller W, Myers EW, Lipman DJ (1990). Basic local alignment search tool. J Mol Biol.

