# Supplementary material for: A modified sequence capture approach allowing standard and methylation analyses of the same enriched genomic DNA sample
Source: BMC Genomics. 2018 Apr 13;19:250. doi: 10.1186/s12864-018-4640-y (PMC5899405; doi:10.1186/s12864-018-4640-y)
Supplement: Supplementary file 1 — Figure S1. Depth of coverage summarised for the non-bisulphite treated samples per extended bait sequence reference contig. Reference extended bait sequence contigs here are organized using POPseq chromosomal pseudomolecules. a) Displays data for the NBTS sample and b) displays data for the NBTF sample. (PDF 1425 kb) [file 12864_2018_4640_MOESM1_ESM.pdf]

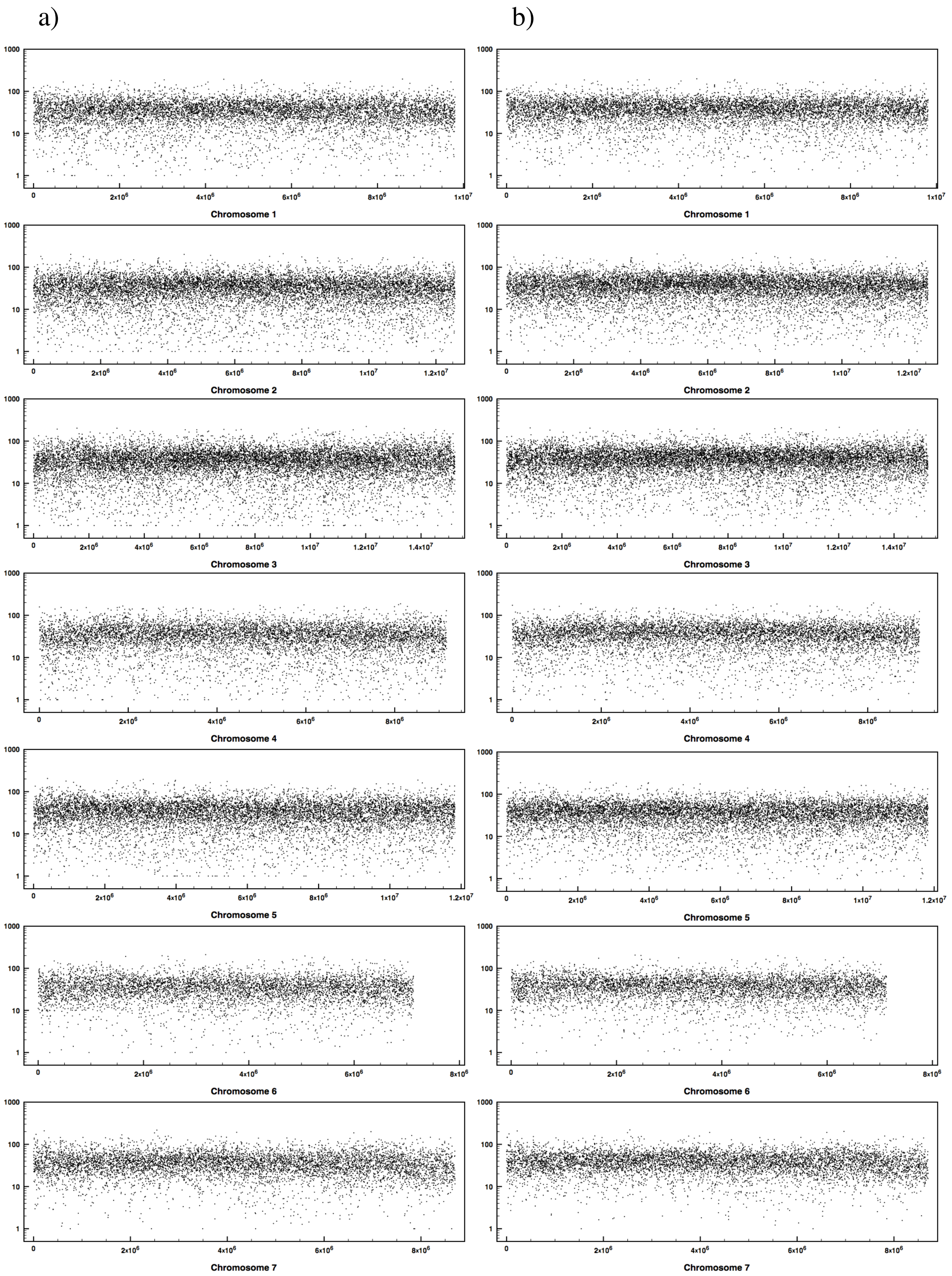

Figure S1 Depth of coverage summarised for the non-bisulphite treated samples per extended bait sequence reference contig. Reference extended bait sequence contigs here are organized using POPseq chromosomal pseudomolecules. a) Displays data for the NBTS sample and b) displays data for the NBTF sample.
