# Supplementary material for: A modified sequence capture approach allowing standard and methylation analyses of the same enriched genomic DNA sample
Source: BMC Genomics. 2018 Apr 13;19:250. doi: 10.1186/s12864-018-4640-y (PMC5899405; doi:10.1186/s12864-018-4640-y)
Supplement: Supplementary file 2 — Figure S2. Design of the 12 Mbp wheat gene capture array. The 110 Mbp design target sequence for the capture probe set is as described by Gardiner et al. (Gardiner et al., 2015). The RNA baits for this SureSelect Methyl-Seq Target Enrichment system are all 120 bp in length, unique, non-repetitive and are evenly placed across the available wheat genic target sequence according to the design illustrated. (PDF 187 kb) [file 12864_2018_4640_MOESM2_ESM.pdf]

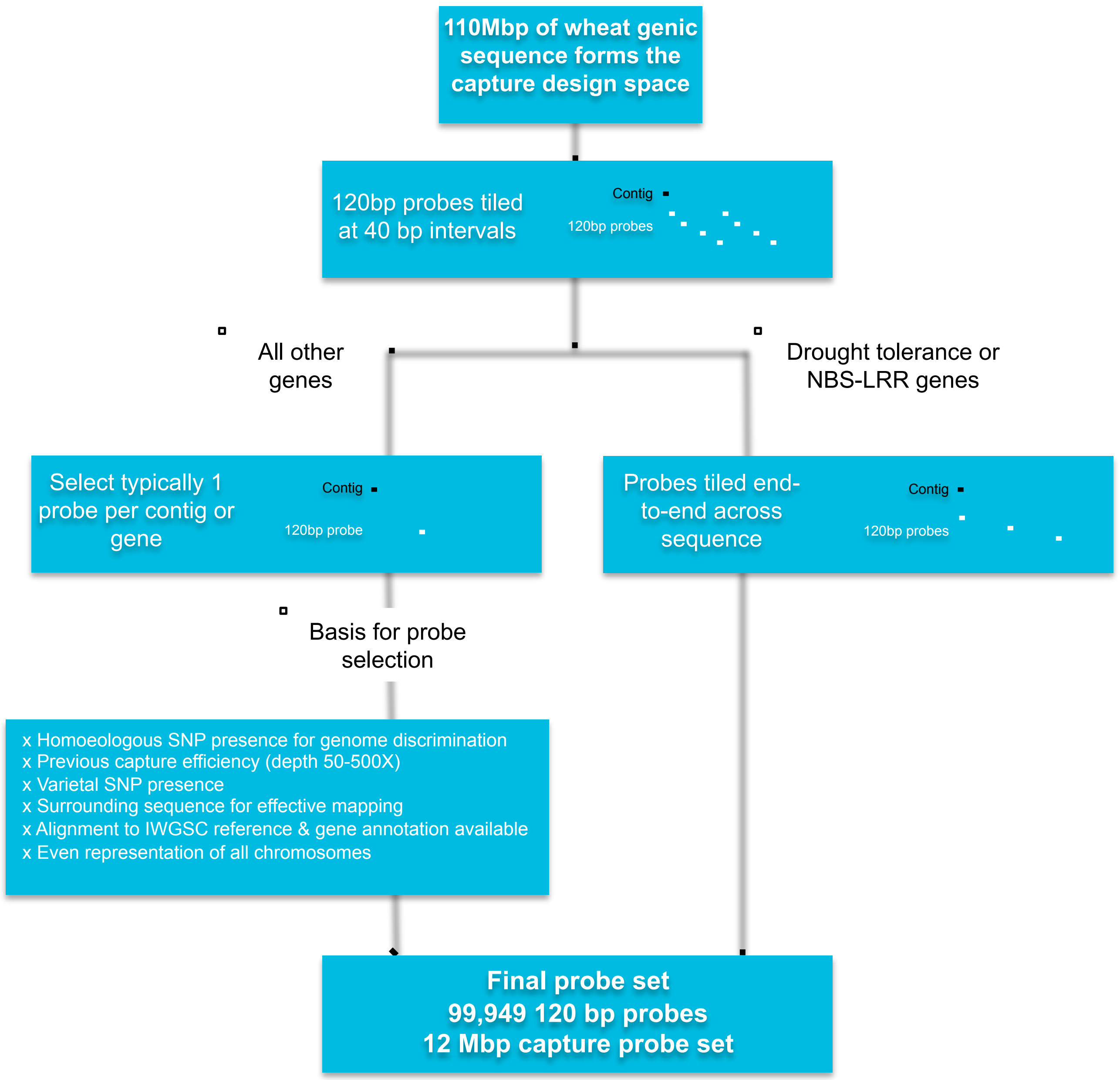

Figure S2. Design of the 12 Mbp wheat gene capture array The 110 Mbp design target sequence for the capture probe set is as described by Gardiner *et al.* (Gardiner *et al.*, 2015). The RNA baits for this SureSelect Methyl-Seq Target Enrichment system are all 120bp in length, unique, non-repetitive and are evenly placed across the available wheat genic target sequence according to the design illustrated.
