# Supplementary material for: A modified sequence capture approach allowing standard and methylation analyses of the same enriched genomic DNA sample
Source: BMC Genomics. 2018 Apr 13;19:250. doi: 10.1186/s12864-018-4640-y (PMC5899405; doi:10.1186/s12864-018-4640-y)
Supplement: Supplementary file 3 — Table S1. Drought tolerance associated genes. 120-mer probes were tiled end-to-end across these genes of particular interest [15–18]. (PDF 81 kb) [file 12864_2018_4640_MOESM3_ESM.pdf]

**Table S1 Drought tolerance associated genes.** 120-mer probes were tiled end-to-end across these genes of particular interest (Liang *et al.,* 2011; Uga *et al.,* 2013; Huang *et al.,* 2008; Hu *et al.,* 2014).

| Gene | Function | Gene | Function |
|---|---|---|---|
| MAPK5 | MAPK-Protein kinase | AT5G52300 (LTI65) | Low temperature induced 65 |
| NPK1 | MAPKKK-Protein kinase | AT5G59220 (PP2C) | Protein phosphatase 2C |
| DSM1 | MAPKKK-Protein kinase | AT5G44420, AT2G26020 (PDF1) | Plant defensin |
| CIPK12 | CBL-interacting protein kinase | AT2G18300 (bHLH) | Basic helix-loop-helix |
| CDPK7 | Calcium-dependent protein kinase | AT2G02990 (RNS1) | Ribonuclease 1 |
| SIK1 | Stress-induced protein kinase (receptor-like kinase) | AT2G47770 | Benzodiazepine receptor-related |
| SOS2 | Serine/threonine kinase | AT1G64110 | AAA-type ATPase family protein |
| DREB (1A, 1B, 1C, 1F, 2, 2A, 2B, 3) | Dehydration responsive element-binding | LEA3/HVA1 | Late embryogenesis abundant protein |
| CBF4 | Dehydration responsive element-binding | NHX1 | Na/H antiporter |
| HARDY | AP2/ERF-like | PIN3t | Auxin efflux carrier |
| bZIP 23,46,72 | bZIP transcription factors | beta | Choline dehydrogenase |
| ABF3 | Abscisic acid responsive elements-binding factor | TsVP | V-H+-PPase |
| AREB1 | bZIP | SRO1c | Similar to RCD1 |
| SNAC1, NAC10, NAC5, NAC6, NAC69 | Stress-induced transcription factor | OAT | Ornithine aminotransferase |
| ZFP252 | C2H2 zinc finger | DRO1 | Deeper rooting 1, early auxin response |
| Zat10 | C2H2-EAR zinc finger | IPT | Isopentenyltransferase |
| MYB2 | MYB domain | TPS1, TPSP | Trehalose-6-phosphate synthase |
| PIMP1 | R2R3 MYB-like protein | P5CS | Delta-1-pyrroline-5-carboxylate synthetase |
| MYB1R | R1-type MYB-like | mtID | Mannitol-1-phosphate dehydrogenase |
| WRKY11/WRKY30 | Transcription factor | RIP18 | Ribosome inactivating protein |
| DIS1, DSG1, DIR1, RDCP1 | E3 ubiquitin ligase | DSM2 | Carotene hydroxylase |
| SQS1 | Squalene synthase | LOS5 | Molybdenum cofactor sulfurase |
| SKIP | Ski-interacting protein | | |
